# Population expectations of primary care quality in 18 countries: a cross-sectional analysis of data from the People’s Voice Survey

**DOI:** 10.1016/j.lanprc.2026.100151

**Published:** 2026-05

**Authors:** Todd P Lewis, Shalom Sabwa, Joao Breda, Susanne Carai, Emma Clarke-Deelder, Svetlana V Doubova, Günther Fink, Patricia J Garcia, Ezequiel Garcia-Elorrio, Hwa-Young Lee, Sailesh Mohan, Inbarani Naidoo, Juhwan Oh, Emelda A Okiro, Ashenif Tadele, Rosanna Tarricone, Marius-Ionuț Ungureanu, Xiaohui Wang, Dong (Roman) Xu, Margaret E Kruk

**Affiliations:** a School of Medicine, Washington University in St Louis, St Louis, MO, USA; b Rollins School of Public Health, Emory University, Atlanta, GA, USA; c WHO Regional Office for Europe, Athens, Greece; d Swiss Tropical and Public Health Institute and University of Basel, Basel, Switzerland; e Epidemiology and Health Services Research Unit, Mexican Institute of Social Security, Mexico City, Mexico; f School of Public Health, Universidad Peruana Cayetano Heredia, Lima, Peru; g Institute for Clinical Effectiveness and Health Policy, Buenos Aires, Argentina; h Graduate School of Public Health and Healthcare Management, The Catholic University of Korea, Seoul, South Korea; i Centre for Chronic Disease Control, New Delhi, India; j Human Sciences Research Council, Pretoria, South Africa; k Seoul National University College of Medicine, Seoul, South Korea; l Population and Health Impact Surveillance Group, Kenya Medical Research Institute–Wellcome Trust Research Programme, Nairobi, Kenya; m Nuffield Department of Medicine, Centre for Tropical Medicine and Global Health, University of Oxford, Oxford, UK; n Health System Research Directorate, Ethiopian Public Health Institute, Addis Ababa, Ethiopia; o Department of Social and Political Science and CERGAS–SDA Bocconi, Bocconi University, Milan, Italy; p Babeş-Bolyai University Cluj-Napoca, Cluj-Napoca, Romania; q Department of Health Management and Policy, School of Public Health, Lanzhou University, Lanzhou, China; r Acacia Lab for Implementation Science, Southern Medical University Institute for Global Health and Center for WHO Studies, School of Health Management and Dermatology Hospital of Southern Medical University, Guangzhou, China

## Abstract

**Background:**

Expectations for health-care quality affect how populations rate primary care, influencing tolerance of poor-quality care and demand for improvement, yet how expectations align with standards of practice remains poorly understood. We aimed to measure population expectations of primary care and their determinants within and across countries.

**Methods:**

In this cross-sectional analysis, we used nationally representative data from the People's Voice Survey collected between May 9, 2022, and Dec 21, 2023, in Ethiopia, Nigeria, Kenya, South Africa, Colombia, Peru, Mexico, Argentina (Province of Mendoza), Uruguay, Laos, India, China, South Korea, Romania, Greece, Italy, the UK, and the USA. Adults aged 18 years and older with at least one health-care visit in the past 12 months were included. Expectations were assessed using two anchoring vignettes, one depicting poor-quality primary care and one depicting adequate-quality primary care, administered via a mobile telephone survey, household interview, or nationally representative web panels. Respondents rated each vignette on a Likert scale: poor, fair, good, very good, or excellent. Low expectations were defined as rating the poor-quality care vignette as fair, good, very good, or excellent, and high expectations were defined as rating the adequate-quality care vignette as poor, fair, or good. Multivariable logistic regression models identified determinants of expectations, including covariates covering demographics, health status, care utilisation and experience, and system competence.

**Findings:**

Among 18 312 respondents (10 000 [54·6%] female and 8312 [45·4%] male; mean age 44 years [SD 17]), 2955 (16·1%) rated poor-quality primary care as fair or better and 11 787 (64·4%) rated adequate-quality primary care as less than very good. The proportion of respondents with low expectations of primary care ranged from 70 (6·3%) of 1112 in the UK to 530 (41·1%) of 1290 in South Korea. The proportion of respondents with high expectations of primary care ranged from 563 (40·1%) of 1404 in Nigeria to 494 (85·0%) of 581 in Italy. Older age, female gender, higher educational attainment, higher income, and use of private care (*vs* public care) were all associated with reduced odds of low expectations. Respondents with higher self-rated health had higher odds of low expectations (*vs* those with poor self-rated health). Activated patients (ie, those who were confident in raising concerns with their provider and that they were responsible for managing their own health) had lower odds of low expectations and lower odds of high expectations (*vs* non-activated patients). And positive perceptions of government management of COVID-19 were associated with high odds of low expectations and low odds of high expectations.

**Interpretation:**

Populations can often misjudge primary care quality, with substantial cross-national variation. Recognising and measuring expectations are essential for accurately interpreting health system ratings and guiding efforts to improve primary care. Policy makers should invest in strategies that strengthen health literacy and patient activation, empowering populations to recognise and demand high-quality primary care.

**Funding:**

Bill & Melinda Gates Foundation, the Swiss Federal Department of Foreign Affairs, Merck Sharp & Dohme, Inter-American Development Bank, the Eckenstein-Geigy Professorship, Initiative on the Future of Health and Economic Resiliency in Africa, National Natural Science Foundation of China, WHO Regional Office for Europe, and the Taejae Foundation.

## Introduction

Many health systems perform below their potential, and primary care—the foundation of most health systems—often underperforms even in routine service delivery.^1^, ^2^, ^3^, ^4^ Poor-quality primary care is characterised by missed or incorrect diagnoses, delayed or inappropriate treatment, suboptimal patient experience, and inadequate follow-up. Strong primary care is associated with better health outcomes, lower costs, and reduced reliance on hospital-based care.^5^ Population feedback on primary care quality is therefore crucial for accountability and improvement, and satisfied users are more likely to seek and adhere to care.^6^

**Research in context**

**Evidence before this study**

We searched PubMed and Google Scholar from Jan 1, 2000, to July 1, 2025. Search terms were combinations of “health systems”, “expectations”, “patient expectations”, “quality of care”, “anchoring vignettes”, and “primary care”. No language restrictions were applied. We also reviewed reference lists of relevant articles and global health system reports. We included studies measuring expectations of health systems or health care and conceptual papers linking expectations to perceptions of health-care quality, satisfaction, or care-seeking. Existing evidence is limited: most studies were single-country or otherwise small in scale, or were not comparable across settings. Only a small number used anchoring vignettes to investigate heterogeneity in ratings of health-care quality.

**Added value of this study**

To our knowledge, this study provides the first nationally representative, multicountry assessment of health system expectations using a harmonised instrument across 18 diverse countries. We quantify expectations of primary care, describe variation within and across countries, and identify groups with systematically low or high expectations—an important dimension for performance measurement and interpreting population-reported care quality. This work extends previous studies, which were limited in sample representativeness or geographical scope, by offering cross-nationally comparable measurement at scale and identifying the factors associated with low and high expectations within populations.

**Implications of all the available evidence**

The combined evidence shows that expectations are a crucial determinant of how people interpret and rate their health system, yet they remain poorly measured and understood. As a result, population ratings that do not account for expectations should be interpreted with caution. Incorporating expectation measures into performance monitoring can clarify where low reported quality reflects genuinely poor performance versus low expectations that might lead patients to be overly satisfied with poor-quality care. For policy makers, these findings highlight the importance of raising expectations alongside improving services to increase demand for high-quality care, while also communicating what the health system can reasonably provide. Future research should track expectations over time and evaluate how expectation-adjusted measures can enhance cross-country benchmarking of health system quality.

User ratings of health system quality are now embedded in several monitoring frameworks.^1^ The WHO Building Blocks Framework identified responsiveness—the health system’s ability to meet the population’s legitimate expectations—as a key goal,^7^ and the *Lancet Global Health* Commission on high-quality health systems emphasised integrating population expectations as foundational to good health systems.^1^

However, population ratings—especially common measures of satisfaction—are shaped by expectations and thus not necessarily objective.^8^^,^^9^ Expectations, central to the provider–patient relationship, are influenced by beliefs, values, personal characteristics, health conditions, and previous experience with care, as well as social and cultural context.^6^^,^^8^^,^^10^ Satisfaction reflects the degree to which pre-existing expectations are met by actual experiences. User feedback is thus difficult to interpret without understanding the expectations that underlie population ratings, particularly when comparing across countries. Few studies have empirically measured expectations, and even fewer allow comparisons across contexts.^6^^,^^11^ Although expectations might not always align with clinical best practice, patients are often well positioned to judge core aspects of quality—such as leaving a visit with a diagnosis and clear advice—without requiring specialised clinical expertise.

Using nationally representative data from the People’s Voice Survey (PVS) in 18 countries, we used ratings of clinical vignettes to systematically measure population expectations of primary care and their determinants within and across countries. We aimed to examine whether populations—and which population subgroups—are demanding too little quality from their care, potentially undermining demand for improvement, or expect more than primary care routinely delivers, which could erode trust in the health system.

## Methods

### Study design and data sources

For this cross-sectional analysis, we used nationally representative data from the PVS collected between May 9, 2022, and Dec 21, 2023, in Ethiopia, Nigeria, Kenya, South Africa, Colombia, Peru, Mexico, Argentina (the Province of Mendoza only), Uruguay, Laos, India, China, South Korea, Romania, Greece, Italy, the UK, and the USA. The survey included samples of 1000 to 2000 adults per country, allowing a margin of sampling error of at most plus or minus 3·1% for full sample estimates.^12^^,^^13^ Interviews and web surveys were conducted by trained interviewers local to each country in collaboration with Ipsos, SSRS, and KStat; in Laos, data collection was managed by an in-house team. The survey was primarily administered via mobile telephone using random-digit dialling; nationally representative web panels were used in South Korea, the UK, and the USA. In Kenya and Ethiopia, additional face-to-face interviews were conducted in rural areas to represent areas with low mobile phone ownership. All adults aged 18 years and older were eligible to participate in the survey; our sample includes respondents who reported at least one health-care visit in the 12 months preceding the survey. Further details on PVS methodology are available elsewhere.^14^

Interviews and web surveys were available in the major languages spoken in each country and consent was obtained verbally in the language of the interview. The Harvard University Institutional Review Board deemed this research exempt from full review (IRB21-0301) and additional local ethical approval was obtained as required in implementing countries.

### Procedures

The PVS uses anchoring vignettes—brief hypothetical scenarios depicting care of varying quality experienced by a fictional patient—to elicit ratings of primary care quality.^15^^,^^16^ The vignettes were developed by an international expert working group and informed by previously tested vignettes.^17^ Content and wording were iteratively refined through expert review and cognitive interviews across all survey languages; the full validation process is described elsewhere.^14^ Respondents rated each vignette on a Likert scale: poor, fair, good, very good, or excellent. For this analysis, we included two primary care vignettes: one illustrating poor quality and one illustrating adequate quality, provided by a hypothetical female primary care physician (appendix p 2). Each was designed to reflect well established signals of primary care quality discernible to respondents—history taking, clinical assessment and treatment, provision of advice, and proposed follow-up.^18^, ^19^, ^20^ Because the vignettes are the same for all respondents, variation in ratings can reveal underlying expectations of care and provides a common measurement benchmark.^16^

We constructed two binary outcome variables to capture misalignment between vignette quality and respondents’ ratings: (1) low expectations—rating the poor-quality vignette as fair, good, very good, or excellent; and (2) high expectations—rating the adequate-quality vignette as poor, fair, or good.

Because the poor-quality vignette was unambiguously deficient, only poor was considered an appropriate response. Both very good and excellent were acceptable ratings for the adequate-quality vignette. We did not accept good as a valid response to the adequate-quality vignette, since it often functions as a neutral or default category rather than a clear endorsement of quality.

**Figure 1:**
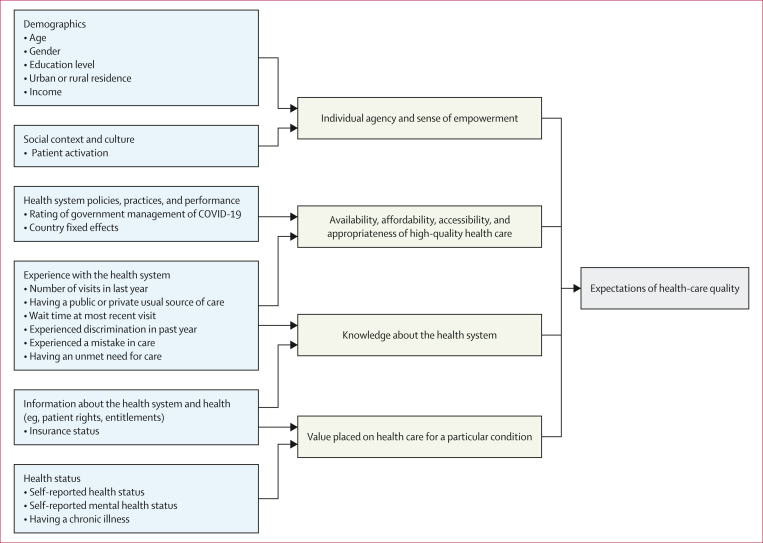
Conceptual framework for expectations of quality with variables measured in the People’s Voice Survey

We developed a framework to conceptualise factors influencing population expectations for care quality, building on work by Roder-DeWan and colleagues^17^ and updated to align with variables measured in the PVS (figure 1).^21^^,^^22^ The framework includes six domains: demographics; social context and culture; health system policies, practices, and performance; experience with the health system; information about the health system and health (eg, patient rights and entitlements); and health status.

Based on this framework, our analysis included data collected via the survey on demographic covariates (ie, age, gender, educational attainment, rural or urban residence, and income tertile), patient activation (defined as being very confident in raising concerns with one’s provider and responsible for managing one’s own health), number of visits in the previous 12 months (ie, more than four *vs* four or fewer), type of usual source of care (ie, private *vs* public), and three system-competence measures (ie, perceived discrimination, perceived medical mistake, and unmet need for care). Gender data were self-reported, with response options of male, female, or another gender (another gender was grouped with female for analysis given small sample sizes). Wait time at the most recent visit (≥1 h) was included as a measure of user experience. Insurance status captured knowledge of patient rights. Health status included self-rated health and mental health (on a Likert scale, poor to excellent) and chronic illness.^23^^,^^24^ Perception of government management of the COVID-19 pandemic (on a Likert scale, poor to excellent) served as a measure of health system performance. Health expenditure per capita was measured using the most recent estimate in international dollars at purchasing power parity from the WHO Global Health Expenditure Database.

### Statistical analysis

We first described respondent characteristics, including the distribution of ratings for both vignettes. We calculated the proportion of respondents with low and high expectations overall, by country, and by gender, and cross-classified ratings to develop profiles of expectations. We plotted low and high expectations against health expenditure per capita and calculated Pearson’s correlation coefficients, with a sensitivity analysis excluding outlier countries.

We constructed two multivariable logistic regression models pooling respondents across countries: one for low expectations and one for high expectations. Both included covariates from our conceptual framework covering demographics, health status, care utilisation and experience, and system competence. We estimated adjusted odds ratios (aORs) for age (continuous), gender (male [ref] *vs* female), educational attainment (none [ref] *vs* primary *vs* secondary *vs* post-secondary), income (lowest [ref] *vs* middle *vs* highest), rurality (urban [ref] *vs* rural), patient activation (not activated [ref] *vs* activated), self-rated health (poor [ref] *vs* fair *vs* good *vs* very good *vs* excellent), self-rated mental health (poor [ref] *vs* fair *vs* good *vs* very good vs excellent), chronic illness (no [ref] *vs* yes), insurance status (uninsured [ref] *vs* insured), usual source of care (public [ref] *vs* private), visit frequency (four or fewer [ref] *vs* more than four visits), wait time at last visit (<1 h [ref] *vs* ≥1 h), unmet need for care (no [ref] *vs* yes), perceived discrimination (no [ref] *vs* yes), perceived medical mistake (no [ref] *vs* yes), and government management of COVID-19 (poor [ref] *vs* fair *vs* good *vs* very good *vs* excellent). We included country fixed effects to account for national-level health system factors, using Ethiopia (a middle performer on both outcomes) as a reference country, and clustered standard errors for in-person interviews in Ethiopia and Kenya. We also estimated unadjusted ORs from bivariate logistic regressions between each covariate and both outcomes. Finally, we conducted a sensitivity analysis of the high expectations model in which only responses of poor or fair were coded as high expectations. Because only 5% of respondents had missing covariate data, analyses were restricted to complete cases.

All descriptive analyses were weighted to reflect the national adult population in each country, with weights normalised to the subset of users. Weights were generated using the latest population reference data and constructed using a raking approach based on age, gender, education, and region. Analyses were performed using Stata version 18.0.

### Role of the funding source

The funders of the study had no role in study design, data collection, data analysis, data interpretation, or writing of the report.

## Results

The PVS surveyed 32 419 adults aged 18 years and older across 18 countries. Of these, 18 312 respondents had used health care in the previous 12 months and had complete covariate data, making them eligible for inclusion (table 1). Among the 18 312 respondents comprising the analytical sample, 10 000 (54·6%) were female, 8312 (45·4%) were male, and the mean age was 44 years (SD 17). Educational attainment was diverse: 1135 (6·2%) had no formal education, 5011 (27·4%) had primary education, 6832 (37·3%) had secondary education, and 5334 (29·1%) had post-secondary education. 5879 (32·1%) lived in rural areas and 13 634 (74·5%) reported having health insurance. 8177 (44·7%) respondents were classified as activated patients.

**Table 1:** Characteristics of health system users in 18 countries

	All participants (n=18 312)
**Demographics**	
Age group, years	
18–39	8367 (45·7%)
40–59	6062 (33·1%)
≥60	3883 (21·2%)
Gender	
Female	10 000 (54·6%)
Male	8312 (45·4%)
Highest educational attainment	
No formal education	1135 (6·2%)
Primary	5011 (27·4%)
Secondary	6832 (37·3%)
Post-secondary	5334 (29·1%)
Income	
Lowest income	7092 (38·7%)
Middle income	6529 (35·7%)
Highest income	4692 (25·6%)
Rural residence	5879 (32·1%)
Activated patient	8177 (44·7%)
**Health status**	
Self-rated health	
Poor	1418 (7·7%)
Fair	4196 (22·9%)
Good	7135 (39·0%)
Very good	3837 (21·0%)
Excellent	1727 (9·4%)
Self-rated mental health	
Poor	579 (3·2%)
Fair	2835 (15·5%)
Good	7032 (38·4%)
Very good	4812 (26·3%)
Excellent	3054 (16·7%)
Chronic illness	6352 (34·7%)
**Care access and utilisation (last 12 months)**	
Insured	13 634 (74·5%)
Private (*vs* public) usual source of care	5396 (29·5%)
High utilisation, >4 visits (*vs* ≤4 visits)	6146 (33·6%)
Long wait at last visit (≥1 h)	4455 (24·3%)
**Health system competence**	
Had unmet need for care	2615 (14·3%)
Perceived discrimination in care	1632 (8·9%)
Perceived medical mistake in care	1687 (9·2%)
Perception of government management of COVID-19 (all time)	
Poor	2686 (14·7%)
Fair	3460 (18·9%)
Good	5592 (30·5%)
Very good	4132 (22·6%)
Excellent	2442 (13·3%)
**Country**	
Ethiopia	1391 (7·6%)
Nigeria	1404 (7·7%)
Kenya	1260 (6·9%)
South Africa	1217 (6·6%)
Colombia	843 (4·6%)
Peru	801 (4·4%)
Mexico	602 (3·3%)
Argentina (Province of Mendoza)	862 (4·7%)
Uruguay	998 (5·4%)
Laos	1082 (5·9%)
India	493 (2·7%)
China	946 (5·2%)
South Korea	1290 (7·0%)
Romania	1466 (8·0%)
Greece	734 (4·0%)
Italy	581 (3·2%)
UK	1112 (6·1%)
USA	1230 (6·7%)
**Health-care vignette ratings**	
Poor-quality primary care vignette	
Poor	15 357 (83·9%)
Fair	1553 (8·5%)
Good	865 (4·7%)
Very good	351 (1·9%)
Excellent	186 (1·0%)
Adequate-quality primary care vignette	
Poor	1596 (8·7%)
Fair	3926 (21·4%)
Good	6265 (34·2%)
Very good	4096 (22·4%)
Excellent	2430 (13·3%)

Data are n (%). Descriptive statistics are weighted to reflect the national population in each country. Analyses are restricted to respondents with complete data on all covariates. Another gender was grouped with female for analysis given small sample sizes. Reported income was divided into tertiles of the distribution of responses. Activation was defined as the respondent being "very confident" they could bring up concerns to their provider and they were the person responsible for managing their own health.

Most respondents had low utilisation of care: of 18 312, 12 166 (66·4%) had four or fewer visits in the past year. 5396 (29·5%) received usual care in the private sector. 1632 (8·9%) reported experiencing discrimination in care and 1687 (9·2%) reported a perceived medical mistake; 4455 (24·3%) waited at least 1 h at their most recent medical visit. Self-rated health was mixed: 5564 (30·4%) reported excellent or very good health, whereas 5614 (30·7%) rated it fair or poor. 6352 (34·7%) reported having a chronic illness.

15 357 (83·9%) of 18 312 respondents correctly rated the poor-quality care vignette as poor (table 1). By contrast, responses to the adequate-quality care vignette were more varied: 6526 (35·6%) of 18 312 rated it as excellent or very good, whereas the remaining 11 787 (64·4%) rated it as less than very good (table 1).

**Figure 2:**
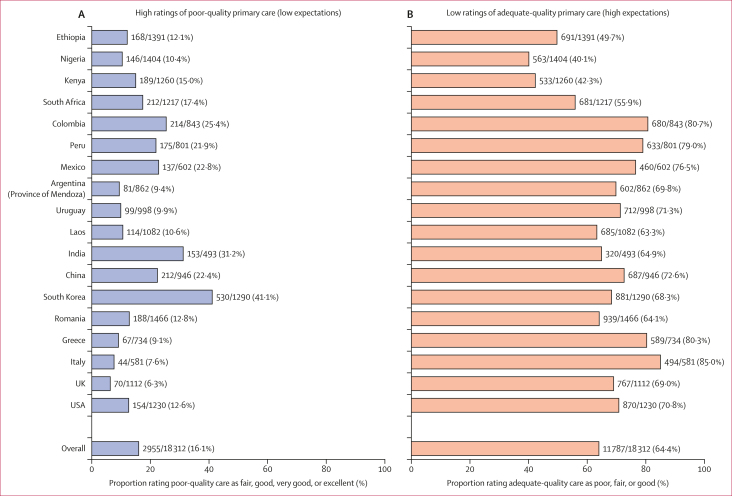
Ratings of poor-quality and adequate-quality primary care vignettes among respondents in 18 countries (A) High ratings of poor-quality care (ie, low expectations). (B) Low ratings of adequate-quality care (ie, high expectations).

On average across countries, 2955 (16·1%) of 18 312 respondents rated the poor-quality care vignette as fair or better, indicating low expectations of care (figure 2). This ranged from 70 (6·3%) of 1112 in the UK to 530 (41·1%) of 1290 in South Korea, with India also high compared with other countries at 154 (31·2%) of 493. By contrast, 11 787 (64·4%) of 18 312 respondents exhibited high expectations based on their ratings of the adequate-quality care vignette, varying from 563 (40·1%) of 1404 in Nigeria and 533 (42·3%) of 1260 in Kenya to 494 (85·0%) of 581 in Italy. Across the two vignettes, 5490 (30·0%) of 18 312 respondents were well aligned with the health system, giving low ratings to poor-quality care and high ratings to adequate-quality care (appendix p 8). 9867 (53·9%) were more demanding, assigning low ratings to both poor-quality and adequate-quality care. Smaller groups included 1036 (5·7%) who were defined as easy to please (ie, gave high ratings for both) and 1919 (10·5%) who were defined as misaligned (ie, gave high ratings for poor-quality care but low ratings for adequate-quality care). There were differences by gender in some countries (appendix p 3).

**Figure 3:**
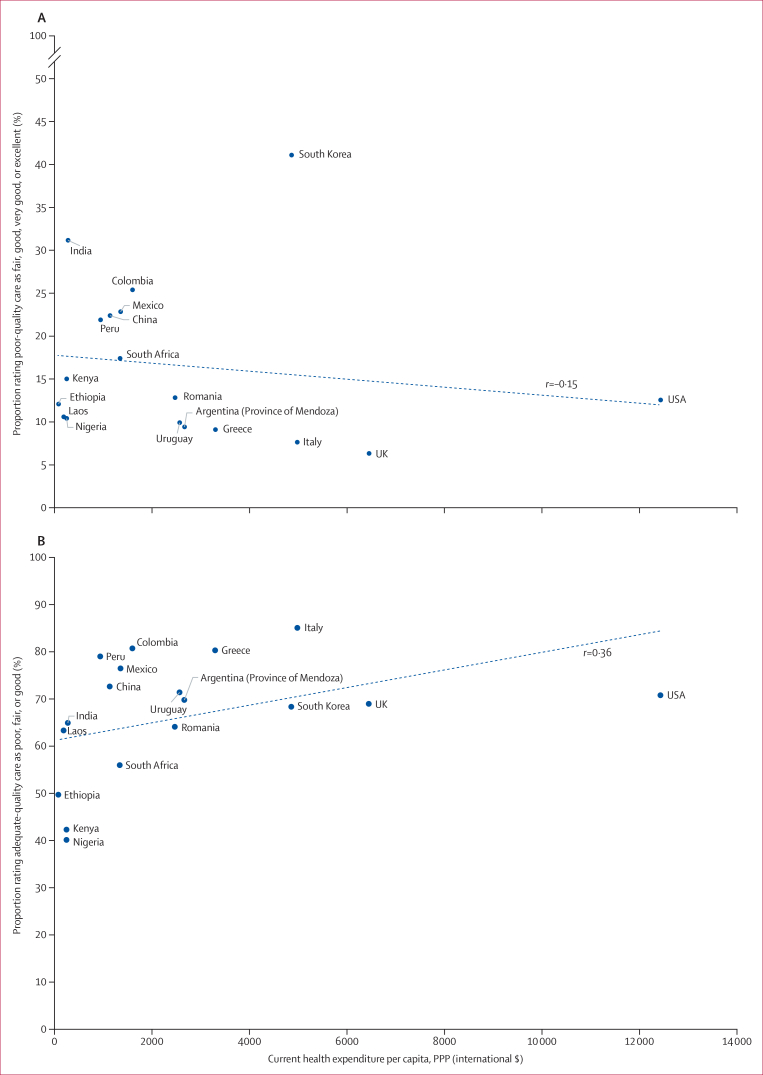
Proportion of respondents with low expectations and high expectations by health expenditure per capita (A) High ratings of poor-quality care by health expenditure (ie, low expectations). (B) Low ratings of adequate-quality care by health expenditure (ie, high expectations). The dashed line indicates the linear trend based on ordinary least squares regression. Note that scales on y axes differ between plots. PPP=purchasing power parity.

Overall, there was virtually no correlation between the proportion of respondents with low expectations and health expenditure per capita (*r*=–0·15; figure 3A). Excluding South Korea and the USA, which were clear outliers, strengthened this relationship (*r*=–0·53). The proportion of respondents with high expectations generally increased with health expenditure, showing a slightly positive correlation (*r*=0·36; *r*=0·49 without the USA; figure 3B).

Results from the first multivariable regression model assessing low expectations of primary care (high ratings of poor-quality care) indicated several significant associations. Older age, female gender, higher educational attainment, and higher income were all associated with reduced odds of low expectations (table 2; unadjusted estimates in appendix pp 4–5). Activated patients had lower odds of low expectations versus inactivated patients. Among care utilisation factors, having a private usual source of care was associated with lower odds of low expectations, while waiting at least 1 h at the last visit was associated with higher odds; no difference was seen by insurance status, high utilisation, or unmet need for care. Compared with those who self-rated their health as poor, respondents reporting their health as very good or excellent had increased odds of low expectations, whereas those who self-reported their health as fair or good showed no difference. Compared with respondents who self-rated their mental health as poor, respondents with good self-rated mental health had increased odds of low expectations, whereas those who self-reported as having fair, very good, or excellent mental health showed no difference. No difference was seen in odds of low expectations by presence of chronic disease or rurality. Better perceptions of government COVID-19 management were associated with increased odds of low expectations versus those with poorer perceptions. No difference in odds of low expectations were seen by perceived discrimination in care or perceived medical mistake in care (table 2).

**Table 2:** Associations between hypothesised determinants and expectations of care in 18 countries

	Low expectations		High expectations	
	aOR (95% CI)	p value	aOR (95% CI)	p value
**Demographics**				
Age (continuous)	0·99 (0·99–0·99)	<0·0001	1·01 (1·01–1·01)	<0·0001
Female (ref: male)	0·69 (0·64–0·75)	<0·0001	1·17 (1·09–1·25)	<0·0001
Educational attainment (ref: none)				
Primary	0·98 (0·73–1·31)	0·90	1·01 (0·79–1·29)	0·93
Secondary	0·74 (0·56–0·99)	0·041	1·02 (0·81–1·29)	0·86
Post-secondary	0·66 (0·49–0·88)	0·0042	1·15 (0·91–1·46)	0·25
Income (ref: lowest income)				
Middle income	0·83 (0·75–0·93)	0·0007	0·97 (0·89–1·05)	0·45
Highest income	0·68 (0·60–0·77)	<0·0001	1·00 (0·91–1·10)	0·92
Rural residence (ref: urban)	1·09 (0·98–1·21)	0·098	0·93 (0·85–1·01)	0·068
Activated patient (ref: not activated)	0·91 (0·83–1·00)	0·049	0·87 (0·81–0·93)	<0·0001
**Health status**				
Self-rated health (ref: poor)				
Fair	1·13 (0·91–1·41)	0·26	0·96 (0·81–1·14)	0·62
Good	1·14 (0·92–1·43)	0·23	0·97 (0·82–1·16)	0·77
Very good	1·39 (1·09–1·76)	0·0074	0·74 (0·61–0·89)	0·0012
Excellent	1·42 (1·09–1·84)	0·0091	0·74 (0·61–0·91)	0·0035
Self-rated mental health (ref: poor)				
Fair	1·30 (0·93–1·82)	0·13	1·22 (0·97–1·55)	0·095
Good	1·47 (1·05–2·04)	0·023	1·33 (1·05–1·67)	0·017
Very good	1·23 (0·88–1·73)	0·23	0·99 (0·79–1·26)	0·965
Excellent	1·24 (0·88–1·75)	0·21	0·98 (0·77–1·25)	0·88
Chronic illness (ref: no chronic illness)	0·96 (0·86–1·07)	0·48	0·94 (0·86–1·02)	0·13
**Care utilisation and experience (last 12 months)**				
Insured (ref: uninsured)	0·91 (0·79–1·04)	0·15	1·07 (0·97–1·19)	0·18
Private usual source of care (ref: public)	0·85 (0·77–0·95)	0·0036	1·02 (0·94–1·11)	0·58
High utilisation, >4 visits (ref: ≤4 visits)	1·00 (0·91–1·11)	0·93	0·88 (0·82–0·95)	0·0012
Wait time at last visit ≥1 h (ref: <1 h)	1·25 (1·13–1·38)	<0·0001	1·00 (0·92–1·09)	0·92
Had unmet need for care (ref: no unmet need)	1·06 (0·92–1·21)	0·41	0·92 (0·83–1·02)	0·12
**Health system competence**				
Perceived discrimination in care (ref: no perceived discrimination)	1·10 (0·94–1·29)	0·25	1·04 (0·92–1·18)	0·53
Perceived medical mistake in care (ref: no perceived medical mistakes)	1·11 (0·95–1·30)	0·17	0·98 (0·87–1·11)	0·80
Perception of government management of COVID-19 (ref: poor)				
Fair	1·57 (1·32–1·86)	<0·0001	0·90 (0·80–1·01)	0·082
Good	2·22 (1·89–2·60)	<0·0001	0·83 (0·74–0·92)	0·0008
Very good	2·32 (1·95–2·75)	<0·0001	0·45 (0·40–0·51)	<0·0001
Excellent	2·38 (1·97–2·87)	<0·0001	0·38 (0·34–0·44)	<0·0001
**Country (ref: Ethiopia)**				
Nigeria	1·13 (0·90–1·41)	0·29	0·37 (0·31–0·43)	<0·0001
Kenya	0·64 (0·50–0·82)	<0·0001	0·55 (0·47–0·65)	<0·0001
South Africa	1·32 (1·06–1·64)	0·013	0·83 (0·70–0·98)	0·024
Colombia	2·40 (1·89–3·04)	<0·0001	2·32 (1·87–2·89)	<0·0001
Peru	1·54 (1·18–1·99)	0·0012	1·88 (1·51–2·34)	<0·0001
Mexico	1·87 (1·44–2·41)	<0·0001	1·93 (1·54–2·43)	<0·0001
Argentina	0·76 (0·57–1·02)	0·071	1·41 (1·15–1·72)	0·0009
Uruguay	0·65 (0·49–0·86)	0·0031	1·94 (1·59–2·35)	<0·0001
Laos	0·76 (0·59–0·99)	0·044	0·95 (0·79–1·13)	0·54
India	2·73 (2·10–3·54)	<0·0001	0·83 (0·66–1·04)	0·10
China	1·60 (1·27–2·01)	<0·0001	1·44 (1·20–1·74)	<0·0001
South Korea	6·33 (5·06–7·90)	<0·0001	1·06 (0·88–1·27)	0·57
Romania	1·49 (1·18–1·90)	0·0010	0·84 (0·70–1·00)	0·051
Greece	0·83 (0·60–1·14)	0·25	2·03 (1·62–2·53)	<0·0001
Italy	0·60 (0·41–0·88)	0·0093	3·08 (2·34–4·06)	<0·0001
UK	0·58 (0·42–0·80)	0·0008	1·07 (0·88–1·30)	0·50
USA	1·46 (1·13–1·89)	0·0037	1·20 (0·99–1·45)	0·062

Models are logistic regressions estimated on an unweighted analytical sample, restricted to respondents with complete data on all covariates. Another gender was grouped with female for analysis given small sample sizes. Reported income was divided into tertiles of the distribution of responses. Activation was defined as the respondent being "very confident" they could bring up concerns to their provider and they were the person responsible for managing their own health. aOR=adjusted odds ratio.

Results from the second multivariable regression model assessing high expectations of primary care (low ratings of adequate-quality care) indicated fewer significant associations. Older age and female gender were associated with increased odds of high expectations (table 2). No difference in odds of high expectations were seen by educational attainment, income, or rurality. Activated patients had lower odds of high expectations than non-activated patients. Among care utilisation factors, respondents with higher utilisation of care (ie, more than four visits) had lower odds of high expectations versus those with four or fewer visits. Compared with respondents with poor self-rated health, respondents with very good or excellent self-rated health had decreased odds of high expectations, and no difference was seen with those with fair or good self-rated health. Compared with respondents with poor self-rated mental health, those with good self-rated mental health had increased odds of high expectations, and no difference was seen among those with fair, very good, or excellent self-rated mental health. No difference in odds of high expectations was seen by chronic illness status. Respondents with good, very good, and excellent perceptions of government COVID-19 management had reduced odds of high expectations compared with those with poor perceptions, and no difference was seen among those who perceived management as fair. No difference in odds of high expectations were seen by perceived discrimination in care or perceived medical mistake in care (table 2).

Compared with Ethiopia, eight countries showed significant associations with odds of both low and high expectations, whereas nine others were associated with either odds of low or high expectations alone (table 2). Adjusting for all covariates, South Korea had the highest odds of low expectations (aOR 6·33, 95% CI 5·06–7·90) and Italy had the highest odds of high expectations (3·08, 2·34–4·06).

Results of the sensitivity analysis, in which high expectations were defined as only a response of poor or fair to the adequate-quality care vignette, were largely consistent with the main findings; however, insurance status and perceiving a medical mistake became significantly associated with odds of high expectations, whereas frequent utilisation was no longer associated with odds of high expectations (appendix pp 6–7).

## Discussion

Analysing population-representative anchoring vignette data from 18 countries, we found that poor-quality primary care was widely recognised; however, 16·1% of respondents classified poor-quality care as fair or higher, indicating diminished expectations. Older age, female gender, higher educational attainment, and higher income were associated with reduced odds of low expectations of primary care, consistent with findings elsewhere.^6^^,^^17^ We also found it was common for people to underestimate the quality of primary care, as 64·4% of respondents rated adequate-quality care as good, fair, or poor. Older age and female gender were associated with increased odds of high expectations, but educational attainment and income were not. Our findings highlight misalignments between what populations expect from primary care and what is defined as adequate quality by standard of practice, with expectations varying by country, individual characteristics, and health system experiences. The results of our analysis are strengthened by use of cross-nationally comparable, nationally representative survey data and by directly measuring misalignment between expectations and quality of care using hypothetical care scenarios. The inclusion of 18 countries spanning regions and national income levels enhances the generalisability of these findings across diverse health system contexts.

Across countries, the proportion of respondents rating poor-quality primary care highly highlights a meaningful segment of the population willing to tolerate poor-quality care. This might reduce accountability and lessen pressure on governments to strengthen primary care systems. Most respondents, however, could identify substandard care. The UK had the lowest proportion of respondents rating poor-quality care favourably, whereas South Korea had the highest proportion, showing that respondents in different settings bring distinct considerations to their expectations of primary care quality. It also challenges assumptions that populations in high-income countries demand more and highlights the role of sociocultural context on expectations; in South Korea, quick, medication-focused visits might align with user preferences despite evidence that patients generally prefer more time with providers.^11^^,^^25^ Similarly, respondents in Colombia, Mexico, and Peru were more likely to tolerate poor-quality care, possibly reflecting historical framing of health systems around principles of solidarity and collective identity.^26^ However, alternative explanations—such as different benchmarks for what constitutes poor-quality care or variation in health literacy—warrant further investigation.

Although most respondents could correctly identify poor-quality care, fewer recognised adequate-quality primary care as such. The high proportion of Italian respondents under-rating adequate-quality care suggests that expectations of service might exceed the requirements or capabilities of effective primary care in Italy. These expectations might reflect a communication gap between the national health system and its citizens, with widespread inadequate health literacy making it difficult to recognise clinical excellence.^27^ Unmet high expectations raise concerns for health systems, as populations that are unable to recognise good-quality primary care might inadvertently demand unrealistic standards, straining resources and leading to unnecessary care. By contrast, all four African countries included in our analysis had comparatively lower proportions of respondents under-rating adequate-quality care, suggesting expectations might be more closely aligned with what primary care can realistically offer.^17^ South Africa stands out, however, as its relatively high health system investment did not translate into higher expectations, possibly reflecting perceived inadequacy of government-provided services.^2^

Cross-classifying responses across the two vignettes yielded four expectation profiles: well aligned, misaligned, easy to please, and more demanding. The large proportion of demanding respondents might reflect how the vignettes’ limited attention to user experience was interpreted as perfunctory care, even when clinical content was adequate. The small but notable easy-to-please group poses a different challenge, as their leniency might reduce accountability for health system quality. The misaligned group could be responding to specific influential cues, such as the receipt of a prescription in the poor-quality care vignette, rather than overall quality. These profiles emphasise the need to tailor communication and service strategies to distinct patient expectations while interpreting survey-based measures of quality with caution.

Previous multicountry work found that over half of respondents rated poor-quality care as good or better.^17^ Our findings show fewer respondents misclassifying poor-quality care, likely due to our vignette design, nationally representative samples, and inclusion of populations with lower education and income.

Patient activation—agency in managing one’s own care and confidence to express concerns—emerged as especially important. Activation was the only factor associated with reduced odds of both low and high expectations, indicating that activated patients are not simply more demanding but appear better able to distinguish poor-quality care from adequate-quality care. This finding highlights patient empowerment as a promising strategy: fostering activation could build legitimate expectations for high-quality care that remain grounded in what systems can reasonably provide.^28^^,^^29^

Experiences with the health system also shaped expectations. Those with a private usual source of care had lower odds of tolerating poor-quality care, perhaps reflecting greater selectivity or exposure to higher quality primary care.^30^ Experiencing long waits might have normalised substandard experiences, as these respondents were more accepting of poor-quality care. Respondents with higher utilisation of care (ie, more than four visits) had lower odds of high expectations, potentially because more frequent use provides more opportunities to judge quality. Respondents who rated government management of the COVID-19 pandemic favourably had higher odds of low expectations and generally lower odds of high expectations, suggesting that general trust in institutions might soften quality judgments.

Expectations were poorly correlated with national health expenditure. Although higher spending is generally associated with better clinical quality, it was weakly associated with populations tolerating poor-quality care.^3^ High expectations showed a stronger correlation with expenditure, suggesting that in higher-spending systems, populations might expect and demand higher-quality primary care.^11^^,^^25^ Expectations thus appear to be embedded in broader societal norms, individual experiences, and trust in institutions, beyond health expenditure alone.

Our analysis has some limitations. Although we used vignettes as measures of expectations for quality of primary care, they captured only one dimension: the perceived clinical quality of a single provider in a basic primary care scenario. Additional vignettes would be needed to assess other domains such as people-centredness. Our vignette design might also have introduced bias. To isolate judgments of technical quality, we excluded user-experience factors (eg, respect, courtesy, timeliness), which might have led respondents to assume a perfunctory visit and contributed to overly demanding patterns. Future designs could address this issue through priming strategies (eg, instructing respondents to assume good customer service and respectful treatment). The physician was depicted as female in both vignettes, which could have influenced ratings through gender bias or unfamiliarity with non-male clinicians. In the adequate-quality vignette, the absence of prescribed medication—often interpreted as a marker of a successful visit—might have reduced ratings and could partly explain the high rates of high expectations. Beyond vignette design, unmeasured influences such as cultural norms, previous experiences, and health literacy could also have affected expectations, although we adjusted for educational attainment as a proxy for health literacy. As with all surveys, interpretations of Likert ratings can vary, and the distance between categories (eg, very good *vs* excellent) might not be equivalent across contexts or individuals, although we sought to mitigate this through careful translation, adaptation, and cognitive interviewing. We suspect several other factors, such as wait time for an appointment, might be associated with high or low expectations but were not measured in the PVS. Finally, the cross-sectional nature of our data precludes inferences about causality.

Our findings highlight that expectations of primary care can often diverge from standards of practice, with some populations either tolerant of poor-quality primary care or dissatisfied with adequate-quality primary care. Low expectations could reduce demand for quality and weaken accountability, while unmet expectations could risk eroding support for health system investments. Differences in expectations also complicate cross-country comparisons, as populations apply fundamentally different benchmarks when rating care. Policy makers should consider strengthening public understanding of primary care quality, communicating standards clearly, and enhancing patient activation to help align expectations with standards of practice.

## Data sharing

All data from the People’s Voice Survey are publicly available at https://dataverse.harvard.edu/dataverse/pvs.

## Declaration of interests

We declare no competing interests.
