# Supplementary material for: Population expectations of primary care quality in 18 countries: a cross-sectional analysis of data from the People’s Voice Survey
Source: Lancet Prim Care. 2026 May;2(5):None. doi: 10.1016/j.lanprc.2026.100151 (PMC13269253; doi:10.1016/j.lanprc.2026.100151)
Supplement: Supplementary appendix [file mmc1.pdf]

THE LANCET
Primary Care

Supplementary appendix

This appendix formed part of the original submission and has been peer reviewed. We post it as supplied by the authors.

Supplement to: Lewis TP, Sabwa S, Breda J, et al. Population expectations of primary care quality in 18 countries: a cross-sectional analysis of data from the People's Voice Survey. *Lancet Prim Care* 2026. https://doi.org/10.1016/j.lanprc.2026.100151

Appendix

Population expectations of primary care quality in 18 countries: A cross-sectional analysis of data from the People's Voice Survey

Table of contents

Appendix box 1: People's Voice Survey anchoring vignettes…………………………………..p.2

Appendix table 1: Ratings of poor and adequate care vignettes among male and female health system users in 18 countries (n=18,312) ………………………………………….................p.3

Appendix table 2: Bivariate associations between hypothesized determinants and expectations of care (n=18,312) …………………………………………………………………………p.4

Appendix table 3: Associations between hypothesized determinants and expectations of care with alternative definition of high expectations (n=18,312) …………………………………………p.6

Appendix figure 1: Profiles of respondent expectations from the People's Voice Survey in 18 countries (n=18,312) ……………………………………………………………..…..p.8

Appendix table 4: Ethical clearance bodies in implementing countries……………………..….p.9

Appendix box 1: People's Voice Survey anchoring vignettes

<table>
<tr><td colspan="2">Now I would like to read you a story to get an understanding of how you rate the quality of care that some people get.

John* has been feeling increasing stomach pain for the past 3 days and decides to go to clinic.</td></tr>
<tr><th>Vignette 1 (poor care)</th><th>Vignette 2 (adequate care)</th></tr>
<tr><td>At the health facility, the doctor does not ask about his symptoms or examine his body; she gives him pain medication and does not give him the diagnosis. How would you rate the quality of care provided?

1. Poor
2. Fair
3. Good
4. Very good
5. Excellent</td><td>Now John* goes to another clinic. There the doctor examines him and orders a blood test. She tells him it is not serious, advises a light diet, and asks him to come back if it gets worse. How would you rate the quality of care provided?

1. Poor
2. Fair
3. Good
4. Very good
5. Excellent</td></tr>
<tr><th>Perceived signals of good or poor medical practice</th><th>Perceived signals of good or poor medical practice</th></tr>
<tr><td>Good practice
• Prescribes treatment

Poor practice
• Does not take history
• Does not conduct examination
• Does not give the diagnosis</td><td>Good practice
• Conducts examination
• Orders diagnostic test
• Provides treatment advice and return guidance

Poor practice
• Does not take history</td></tr>
</table>

*Name adapted for local relevance in each country.

Appendix table 1: Ratings of poor and adequate care vignettes among male and female health system users in 18 countries (n=18,312)

	Female		Male	
**Country**	**Low expectations, n/N (%)**	**High expectations, n/N (%)**	**Low expectations, n/N (%)**	**High expectations, n/N (%)**
Argentina (Mendoza)	47/558 (8.5)	386/558 (69.2)	34/304 (11.1)	216/304 (70.9)
China	81/479 (16.8)	338/479 (70.6)	131/466 (28.1)	348/466 (74.7)
Colombia	109/468 (23.2)	373/468 (79.7)	105/374 (28.0)	307/374 (82.0)
Ethiopia	68/734 (9.3)	299/734 (40.7)	100/657 (15.2)	392/657 (59.7)
Greece	18/407 (4.3)	348/407 (85.5)	49/327 (15.1)	241/327 (73.6)
India	66/226 (29.3)	153/226 (67.7)	87/266 (32.8)	166/266 (62.5)
Italy	19/304 (6.3)	272/304 (89.3)	25/277 (9.1)	222/277 (80.4)
Kenya	85/718 (11.9)	276/718 (38.4)	104/542 (19.1)	257/542 (47.4)
Lao PDR	52/600 (8.7)	389/600 (64.8)	62/482 (13.0)	296/482 (61.4)
Mexico	73/321 (22.7)	258/321 (80.6)	64/281 (22.9)	202/281 (71.8)
Nigeria	81/757 (10.7)	342/757 (45.2)	65/648 (10.0)	221/648 (34.1)
Peru	79/441 (17.8)	364/441 (82.6)	97/360 (26.9)	268/360 (74.6)
Republic of Korea	254/678 (37.4)	502/678 (74.0)	276/611 (45.1)	379/611 (62.1)
Romania	88/810 (10.8)	541/810 (66.8)	100/656 (15.3)	398/656 (60.7)
South Africa	113/715 (15.8)	402/715 (56.2)	99/502 (19.7)	279/502 (55.6)
United Kingdom	33/582 (5.6)	419/582 (72.0)	38/530 (7.1)	348/530 (65.6)
United States	79/650 (12.1)	487/650 (74.9)	76/580 (13.1)	384/580 (66.2)
Uruguay	51/551 (9.2)	401/551 (72.8)	48/447 (10.8)	311/447 (69.5)

Notes: Low expectations defined as rating poor-quality vignette as fair or better. High expectations defined as rating adequate-quality vignette below very good.

Appendix table 2: Bivariate associations between hypothesized determinants and expectations of care (n=18,312)

	Low expectations		High expectations	
**Variable**	**OR**	**95% CI**	**OR**	**95% CI**
**Demographics**				
Age (continuous)	**0.99**	**0.99–1.00**	**1.01**	**1.01–1.02**
Female	**0.70**	**0.62–0.79**	**1.12**	**1.00–1.24**
Educational attainment (ref: None)				
Primary	1.12	0.73–1.72	1.29	0.87–1.93
Secondary	1.06	0.67–1.67	1.43	0.97–2.13
Post-secondary	1.09	0.70–1.70	**1.73**	**1.17–2.57**
Income (ref: Lowest income)				
Middle income	**0.85**	**0.74–0.98**	1.11	0.95–1.29
Highest income	**0.75**	**0.63–0.90**	**1.18**	**1.01–1.38**
Rural residence (ref: Urban)	1.02	0.86–1.20	**0.62**	**0.53–0.73**
Activated patient	**0.76**	**0.66–0.88**	**0.72**	**0.65–0.81**
**Health status**				
Self-rated health (ref: Poor)				
Fair	1.15	0.86–1.53	1.13	0.87–1.46
Good	1.04	0.78–1.38	1.11	0.86–1.43
Very Good	1.08	0.79–1.48	**0.63**	**0.48–0.83**
Excellent	1.10	0.80–1.52	**0.49**	**0.37–0.65**
Self-rated mental health (ref: Poor)				
Fair	1.37	0.86–2.19	1.17	0.80–1.71
Good	1.43	0.91–2.26	1.20	0.85–1.70
Very Good	1.26	0.78–2.04	**0.66**	**0.46–0.95**
Excellent	1.29	0.82–2.05	**0.66**	**0.46–0.96**
Chronic illness	0.87	0.75–1.01	**1.20**	**1.07–1.35**
**Care utilization and experience (last 12 months)**				
Insured	1.02	0.85–1.23	**2.27**	**1.96–2.63**
Private usual source of care	1.13	0.99–1.29	0.98	0.87–1.10
Visits (more than 4)	1.01	0.90–1.15	**1.12**	**1.00–1.25**
Wait time at last visit 1 hour or longer	**1.30**	**1.11–1.54**	0.98	0.86–1.12
Had unmet need for care	0.90	0.72–1.11	0.89	0.74–1.07
**Health system competence**				
Perceived discrimination in care	1.10	0.88–1.38	0.92	0.74–1.15
Perceived medical mistake in care	1.10	0.92–1.31	0.93	0.77–1.12
Government management of COVID-19 (ref: Poor)				
Fair	**2.10**	**1.68–2.62**	1.08	0.91–1.27

Good	**2.46**	**1.98–3.04**	1.02	0.87–1.19
Very Good	**2.81**	**2.17–3.66**	**0.42**	**0.35–0.51**
Excellent	**2.58**	**1.99–3.34**	**0.37**	**0.30–0.46**
Country (ref: Ethiopia)				
Nigeria	0.84	0.52–1.37	0.68	0.42–1.09
Kenya	1.28	0.49–3.39	0.74	0.40–1.37
South Africa	**1.53**	**1.04–2.27**	1.29	0.86–1.92
Colombia	**2.47**	**1.72–3.55**	**4.24**	**2.79–6.44**
Peru	**2.04**	**1.34–3.10**	**3.81**	**2.44–5.97**
Mexico	**2.15**	**1.47–3.15**	**3.29**	**2.16–5.01**
Argentina	0.76	0.51–1.12	**2.34**	**1.59–3.45**
Uruguay	0.80	0.53–1.20	**2.52**	**1.70–3.75**
Laos	0.86	0.54–1.37	**1.75**	**1.15–2.65**
India	**3.29**	**2.12–5.10**	**1.88**	**1.19–2.95**
China	**2.10**	**1.39–3.17**	**2.69**	**1.76–4.10**
South Korea	**5.07**	**3.67–7.01**	**2.19**	**1.50–3.18**
Romania	1.07	0.73–1.56	**1.81**	**1.22–2.67**
Greece	0.73	0.45–1.17	**4.13**	**2.61–6.52**
Italy	**0.60**	**0.36–0.99**	**5.76**	**3.67–9.04**
United Kingdom	**0.49**	**0.32–0.75**	**2.25**	**1.54–3.31**
United States	1.04	0.72–1.51	**2.45**	**1.67–3.60**

Notes: Low expectations defined as rating poor-quality vignette as fair or better. High expectations defined as rating adequate-quality vignette below very good. Bolded values indicate statistical significance at the $p<0.05$ level. Reference categories shown in parentheses.

Appendix table 3: Associations between hypothesized determinants and expectations of care with alternative definition of high expectations (n=18,312)

Variable	High expectations	
	aOR	95% CI
**Demographics**		
Age	1.00	1.00–1.00
Female	**1.07**	**1.00–1.15**
Educational attainment (ref: None)		
Primary	1.15	0.89–1.49
Secondary	1.10	0.86–1.42
Post-secondary	1.11	0.86–1.43
Income (ref: Lowest income)		
Middle income	1.01	0.92–1.10
Highest income	0.95	0.86–1.05
Rural residence (ref: Urban)	1.00	0.91–1.09
Activated patient	**0.84**	**0.78–0.90**
**Health status**		
Self-rated health (ref: Poor)		
Fair	0.90	0.77–1.05
Good	**0.78**	**0.66–0.91**
Very Good	**0.66**	**0.55–0.79**
Excellent	**0.70**	**0.57–0.86**
Self-rated mental health (ref: Poor)		
Fair	1.17	0.93–1.48
Good	1.04	0.83–1.30
Very Good	0.99	0.79–1.25
Excellent	1.11	0.88–1.40
Chronic illness	0.93	0.85–1.01
**Care utilization and experience (last 12 months)**		
Insured	**1.16**	**1.03–1.30**
Private usual source of care	1.08	0.99–1.18
Visits (more than 4)	0.96	0.89–1.03
Wait time at last visit 1 hour or longer	0.98	0.90–1.07
Had unmet need for care	0.92	0.83–1.02
**Health system competence**		
Perceived discrimination in care	1.12	0.99–1.27
Perceived medical mistake in care	**1.13**	**1.00–1.27**
Government management of COVID-19 (ref: Poor)		
Fair	**0.87**	**0.78–0.97**
Good	**0.60**	**0.54–0.66**
Very Good	**0.57**	**0.51–0.64**
Excellent	**0.57**	**0.50–0.65**

**Country (ref: Ethiopia)**		
Nigeria	**0.29**	**0.24–0.37**
Kenya	**0.53**	**0.43–0.65**
South Africa	1.04	0.87–1.24
Colombia	**1.42**	**1.17–1.74**
Peru	**1.25**	**1.03–1.53**
Mexico	**1.49**	**1.21–1.83**
Argentina	0.93	0.75–1.14
Uruguay	1.05	0.86–1.28
Laos	**0.54**	**0.44–0.66**
India	**0.64**	**0.49–0.84**
China	**1.30**	**1.08–1.56**
South Korea	0.92	0.76–1.12
Romania	**0.60**	**0.50–0.73**
Greece	**1.42**	**1.16–1.75**
Italy	**2.33**	**1.87–2.92**
United Kingdom	**1.24**	**1.02–1.51**
United States	1.13	0.93–1.37

Notes: Models are logistic regressions estimated on an unweighted analytic sample, restricted to respondents with complete data on all covariates. In contrast to the main model, which defines high expectations as ratings of “poor,” “fair,” or “good” to the adequate care vignette, high expectations here excludes “good” and includes ratings of “poor” or “fair” only. Reported income was divided into tertiles of the distribution of responses. Activation was defined as being "very confident" you could bring up concerns to your provider and you were the person responsible for managing your own health. Bolded values indicate statistical significance at the $p<0.05$ level.

Appendix figure 1: Profiles of respondent expectations from the People's Voice Survey in 18 countries (n=18,312)

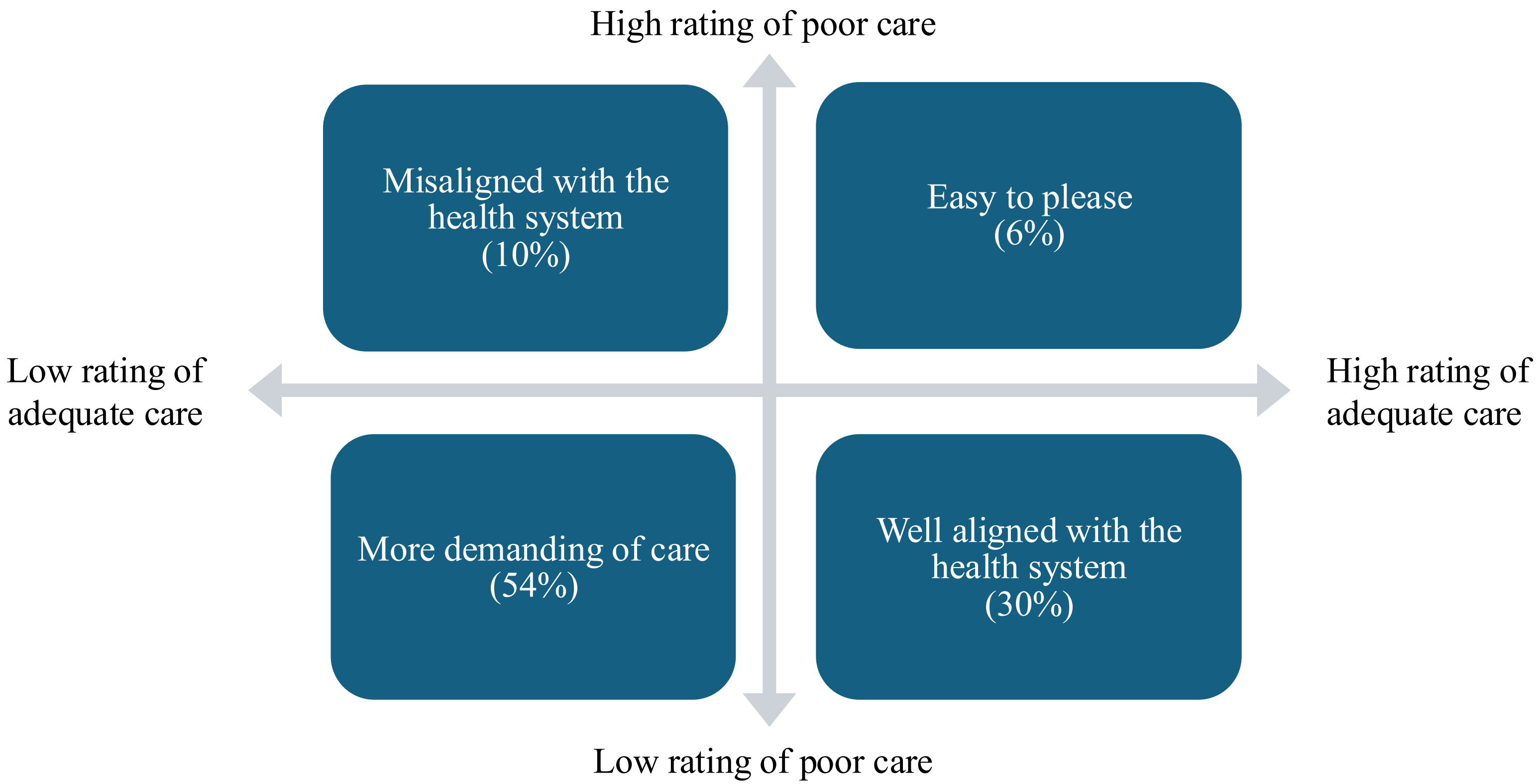

Appendix table 4: Ethical clearance bodies in implementing countries

Country	Ethical clearance bodies
Argentina	Provincial Council for Research Ethics Evaluation, Mendoza; Harvard Longwood Campus Institutional Review Board
China	Ethical Review Board, School of Public Health, Lanzhou University; Harvard Longwood Campus Institutional Review Board
Colombia	Research Ethics Committee of the Central Military Hospital, Colombia; Harvard Longwood Campus Institutional Review Board
Ethiopia	Ethiopian Public Health Institute Institutional Review Board; Harvard Longwood Campus Institutional Review Board
Greece	WHO Research Ethics Review Committee; Harvard Longwood Campus Institutional Review Board
India	India Centre for Media Studies (CMS); Harvard Longwood Campus Institutional Review Board
Italy	Harvard Longwood Campus Institutional Review Board
Kenya	KEMRI Scientific and Ethics Review Unit (SERU); National Commission for Science, Technology and Innovation (NACOSTI); Harvard Longwood Campus Institutional Review Board
Laos	National Ethics Committee for Health Research (NECHR); Harvard Longwood Campus Institutional Review Board
Mexico	Harvard Longwood Campus Institutional Review Board
Nigeria	Harvard Longwood Campus Institutional Review Board
Peru	Institutional Research Ethics Committee of Cayetano Heredia University; Harvard Longwood Campus Institutional Review Board
Romania	National Institute of Public Health (Institutul Național de Sănătate Publică); Harvard Longwood Campus Institutional Review Board
South Africa	UKZN Biomedical Research Ethics Committee; Harvard Longwood Campus Institutional Review Board
South Korea	Seoul National University Hospital Institutional Review Board; Harvard Longwood Campus Institutional Review Board
United Kingdom	Harvard Longwood Campus Institutional Review Board
United States	Harvard Longwood Campus Institutional Review Board
Uruguay	Harvard Longwood Campus Institutional Review Board
